# Tissue dyslipidemia in salmonella-infected rats treated with amoxillin and pefloxacin

**DOI:** 10.1186/1476-511X-11-152

**Published:** 2012-11-09

**Authors:** Solomon O Rotimi, David A Ojo, Olusola A Talabi, Elizabeth A Balogun, Oladipo Ademuyiwa

**Affiliations:** 1 Biochemistry Unit, Department of Biological Sciences, Covenant University, Ota, Nigeria; 2 Department of Microbiology, University of Agriculture, Abeokuta, Nigeria; 3 Medical Centre, University of Agriculture, Abeokuta, Nigeria; 4 Department of Biochemistry, University of Agriculture, Abeokuta, Nigeria

**Keywords:** Salmonellosis, Cholesterogenesis, Phospholipidosis, Amoxicillin, Pefloxacin

## Abstract

**Background:**

This study investigated the effects of salmonella infection and its chemotherapy on lipid metabolism in tissues of rats infected orally with *Salmonella typhimurium* and treated intraperitoneally with pefloxacin and amoxillin.

**Methods:**

Animals were infected with *Salmonella enterica* serovar *Typhimurium* strain TA 98. After salmonellosis was confirmed, they were divided into 7 groups of 5 animals each. While one group served as infected control group, three groups were treated with amoxillin (7.14 mg/kg body weight, 8 hourly) and the remaining three groups with pefloxacin (5.71mg/kg body weight, 12 hourly) for 5 and 10 days respectively. Uninfected control animals received 0.1ml of vehicle. Rats were sacrificed 24h after 5 and 10 days of antibiotic treatment and 5 days after discontinuation of antibiotic treatment. Their corresponding controls were also sacrificed at the same time point. Blood and tissue lipids were then evaluated.

**Results:**

Salmonella infection resulted in dyslipidemia characterised by increased concentrations of free fatty acids (FFA) in plasma and erythrocyte, as well as enhanced cholesterogenesis, hypertriglyceridemia and phospholipidosis in plasma, low density lipoprotein-very low density lipoprotein (LDL-VLDL), erythrocytes, erythrocyte ghost and the organs. The antibiotics reversed the dyslipidemia but not totally. A significant correlation was observed between fecal bacterial load and plasma cholesterol (r=0.456, p<0.01), plasma triacyglycerols (r=0.485, p<0.01), plasma phospholipid (r=0.414, p<0.05), plasma free fatty acids (r=0.485, p<0.01), liver phospholipid (r=0.459, p<0.01) and brain phospholipid (r=0.343, p<0.05).

**Conclusion:**

The findings of this study suggest that salmonella infection in rats and its therapy with pefloxacin and amoxillin perturb lipid metabolism and this perturbation is characterised by cholesterogenesis.

## Background

*Salmonella spp.* are important aetiological agents of various diseases resulting from feco-oral infection. A characteristic feature of the organisms in the genus Salmonella is their wide host range which comprises most animal species including mammals, birds and cold-blooded animals [1]. There are two types of food-borne infections causing serious medical and veterinary problems worldwide: i) intestinal or non-typhoid form (salmonellosis) caused by hundreds of different serovariants of *S. enterica*, including serovar Typhimurium and ii) generalized or typhoid form (enteric fever) caused by *S. enterica* serovar Typhi. Enterocolitis characteristic of non-typhoid forms is by far the most common manifestation of salmonella-derived infections in humans that under certain circumstances are accompanied by septicaemia [2,3], causing the increase of mortality.

By two distinct mechanisms (via M cells or enterocytes) *S. typhi* is believed to translocate rapidly and efficiently from the lumen of the human intestine through the mucosa to eventually reach the reticuloendothelial system and gall bladder, where, following a relatively long incubation, they precipitate a systemic febrile illness accompanied by a low-level secondary bacteremia [1,4] often resulting in sepsis [5-9].

The host metabolic response of the body following sepsis comprises mobilization of substrates from the periphery to be utilized by visceral tissues and immune cells [10], resulting in loss of lean body mass. Consequently, gluconeogenic and other amino acids are released in increased amounts into the circulation. The glucose produced in this response is the main energy source for cells involved in the host immune response during sepsis [10]. In addition to accelerated gluconeogenesis, peripheral mobilized amino acids serve as substrates for central organs, including liver (for acute-phase protein (APR) synthesis) and enterocytes [11,12]. Although, changes in carbohydrate metabolism with consecutive hyperglycemia and hyperlactatemia, and in protein metabolism, which results in enhanced acute-phase protein synthesis and tissue protein catabolism with subsequent negative nitrogen balance have been reported, there is a dearth of information on the lipid metabolism in the tissues of the host.

As a result of these alterations and their consequence on the host, the management of salmonellosis and/or the resulting sepsis often involves supportive measures as well as the use of antibiotics which are generally employed as the first-line treatment [9,13,14]. Fluoroquinolones (e.g. pefloxacin) are considered to be optimal therapy while amoxillin is often used as alternative effective therapy [13-15]. Amoxillin is a β-lactam aminopenicillin and acts by inhibiting the synthesis of bacterial cell walls while pefloxacin as a fluoroquinolone blocks bacterial DNA synthesis [1,2]. Despite the use of antibiotics, aggressive operative intervention, nutritional support, and even anticytokine therapies, multiple organ failure continues to be a major cause of morbidity and mortality in sepsis [1,15-17]. Thus, it is essential to study the impact of chemotherapy on tissue lipid metabolism in experimental salmonellosis.

## Results

Table 1 summarises the effects of pefloxacin and amoxillin on the fecal bacteria load and weight gain of the rats infected with *S. typhimurium*. The oral infection of the rats with *S. typhimurium* caused salmonellosis as observed by the apperance of *S. typhimurium* in the feaces of the rats. The concentration of *S. typhimurium* cultured in the feaces was significantly (p<0.05) decreased upon the administration of pefloxacin and amoxillin for 5 days. The decrease was sustained even 5 days after discontinuing antibiotic administration. The body weight of the animals was significantly (p<0.05) decreased in *S. typhimurium* infection. Although the drugs reversed the weight loss, none was able to bring it to control values.

**Table 1:** Fecal bacteria load and weight gain of the animals

	**Normal control**	**Infection control**	**Infected+ pefloxacin day 5**	**Infected+ pefloxacin day 10**	**Infected + pefloxacin day 15**	**Infected+ amoxillin day 5**	**Infected+ amoxillin day 10**	**Infected + amoxillin day 15**
Fecal bacteria load (log CFU/g feaces)	0.00±0.00^a^	4.34±0.01^d^	4.03±0.01^c^	2.59±0.24^b^	2.31±0.09^b^	4.11±0.06^c^	2.99±0.08^b^	2.64±0.04^b^
Weight change (g)	32.80±4.41^a^	−14.33±2.39^d^	−1.00±1.00^c^	−4.00±4.15^c^	−8.50±3.37^c^	0.00±1.67^b^	−7.50±7.98^c^	−2.50±1.80^c^

Each value represents the mean±S.E.M of 5 rats. Values within the same row with different superscripts are significantly different at p<0.05.

In Table 2 is the summary of the effects of pefloxacin and amoxillin on plasma, HDL and LDL-VLDL lipid profiles of rats infected with *S. typhimurium*. Salmonellosis caused a double-fold increase in the plasma level of cholesterol, triacylglycerols, phospholipid and free fatty acids. The increase in cholesterol level was reversed by treatment with pefloxacin and amoxillin for 5 days and 10 days. On day 15, only amoxillin had a further significant (p<0.05) reduction; however, this was still significatly (p<0.05) higher than the cholesterol in the plasma of the normal control. The increase in triacylglycerol was reversed more by pefloxacin on day 5 and day 10 than amoxillin. This trend continued to day 15 with animals infected pefloxacin day 15 group having triacylglycerol level not significantly (p>0.05) different from the normal control. After 5 days of treating the infected animals, only pefloxacin significantly (p<0.05) reduced the level of phospholipids in the plasma. However, upon completion of the treatments on day 10, both pefloxacin and amoxillin had produced a similar reduction of the elevated plasma phospholipid level. Although, no further reduction was observed in animals in infected pefloxacin day 15 group, that of the infected animals treated with amoxillin was reversed to a level not significantly (p>0.05) different from the normal control on day 15.

**Table 2:** Plasma, HDL and LDL-VLDL lipid profile of the animals

**Plasma**	**Normal control**	**Infection control**	**Infected+ pefloxacin day 5**	**Infected+ pefloxacin day 10**	**Infected + pefloxacin day 15**	**Infected+ amoxillin day 5**	**Infected+ amoxillin day 10**	**Infected + amoxillin day 15**
Cholesterol (mg/dl)	56.60±2.43^a^	104.13±6.60^b^	79.14±5.49^c^	79.04±5.84^c^	77.96±5.73^c^	59.86±7.54^d^	73.55±7.05^c^	69.16±6.70^d^
Triacylglycerols (mg/dl)	63.18±5.19^a^	125.18±14.26^c^	77.32±0.99^b^	97.48±10.89^c^	58.43±5.89^a^	112.78±6.78^d^	127.58±17.61^d^	68.16±3.72^b^
Phospholipid (mg/dl)	75.33±15.41^a^	129.60±23.13^c^	122.96±7.93^c^	100.60±15.55^b^	109.84±10.18^b^	130.25±18.29^c^	109.35±10.40^b^	66.10±20.74^a^
Free Fatty acid (mg/dl)	14.10±2.25^a^	23.08±0.75^c^	17.20±0.39^b^	16.50±1.23^b^	17.28±0.55^b^	17.70±1.18^b^	16.16±0.53^b^	18.87±0.74^b^
**HDL**								
Cholesterol (mg/dl)	39.06±2.77^b^	22.19±0.94^a^	21.08±4.03^a^	37.19±8.41^b^	21.16±1.39^a^	18.06±5.97^a^	36.76±2.74^b^	22.36±1.75^a^
Triacylglycerols (mg/dl)	26.83±3.65^a^	37.75±7.17^a^	25.12±3.63^a^	33.98±3.43^a^	25.81±2.29^a^	28.26±5.51^a^	27.28±4.08^a^	39.50±4.14^a^
Phospholipid (mg/dl)	68.53±1.42^a^	29.57±1.71^b^	89.91±19.50^e^	73.87±12.24^d^	52.49±7.31^c^	71.93±7.31^d^	66.10±14.67^d^	48.60±7.37^c^
**LDL-VLDL**								
Cholesterol (mg/dl)	25.94±2.31^b^	49.03±2.39^e^	15.24±2.32^a^	43.38±3.75^e^	36.17±3.23^d^	28.64±3.34^c^	43.38±3.00^e^	34.33±3.80^d^
Triacylglycerols (mg/dl)	22.43±1.72^a^	68.66±4.26^d^	24.02±1.40^a^	56.50±8.71^c^	36.90±1.58^b^	28.61±2.78^a^	49.05±3.57^c^	58.37±7.38^d^
Phospholipid (mg/dl)	86.99±2.81^a^	95.18±5.63^c^	64.64±4.04^b^	110.81±2.73^c^	107.41±2.14^c^	69.50±4.18^b^	117.13±9.23^d^	83.11±4.31^a^

Each value represents the mean±S.E.M of 5 rats. Values within the same row with different superscripts are significantly different at p<0.05.

Salmonellosis caused a significant (p<0.05) decrease in the level of HDL cholesterol and HDL phospholipid whereas no significant change was observed in the HDL triacylglycerol level. Treatment with the antibiotics reversed the trend observed in cholesterol and phospholipid, although at 5 days post-treatment with amoxillin, HDL phospholipid was about 71% of the control value.

Salmonellosis also resulted in a significant (p<0.05) increase in the levels of cholesterol, triacylglycerols and phospholipid in the LDL-VLDL fraction of the plasma. Although the increase in the cholesterol and triacylglycerols concentrations was reversed by pefloxacin and amoxillin on day 5, a rebound to higher values was observed on day 10 for both drugs. By day 15 however, a significant drop was observed with values obtained for both drugs being significantly higher than controls (p<0.05) for phospholipid, however, the rebound to higher values was sustained till day 15 in pefloxacin-treated animals, whereas a decrease on day 15 was observed in amoxillin-treated animals.

Table 3 summarises the effects of pefloxacin and amoxillin on erythrocyte and erythrocyte ghost lipid profiles of rats infected with *S. typhimurium*. While Salmonellosis caused a significant (p<0.05) increase in the erythrocyte cholesterol, phospholipid and free fatty acid levels with a significant (p<0.05) decrease in triacylglyceride level, the increase in the cholesterol concentration in the erythrocyte was reversed by pefloxacin on day 5 while both drugs significantly (p<0.05) reduced it to a level significantly (p<0.05) lower than the normal control by day 10. On day 15, rats treated with amoxillin had a significantly (p<0.05) increased cholesterol concentration to a level higher than the infection control. There was a further decrease in the level of erythrocyte triacylglycerols on day 5 in the rats treated with both pefloxacin and amoxillin. This was however reversed with amoxillin to a level not significantly (p>0.05) different from normal control and by pefloxacin to a level significantly (p<0.05) higher than normal control. Pefloxacin on day 5 and day 10 reversed the increase in the level of phospholipid in the erythrocyte. Rats treated with amoxillin had their erythrocyte phospholipid level significantly (p<0.05) higher on day 10 than normal control. Although both antibiotics on day 5 and pefloxacin on day 10 reversed the salmonellosis-induced increased free fatty acid, values obtained were still significantly higher than normal control. Values obtained with amoxillin on day 10 and pefloxacin on day 15 were not significantly (p>0.05) different from normal control.

**Table 3:** Erythrocyte and erythrocyte ghost lipid profile of the animals

**Erythrocyte**	**Normal control**	**Infection control**	**Infected+ pefloxacin day 5**	**Infected+ pefloxacin day 10**	**Infected + pefloxacin day 15**	**Infected+ amoxillin day 5**	**Infected+ amoxillin day 10**	**Infected + amoxillin day 15**
Cholesterol (mg/dl)	113.43±19.54^a^	127.20±11.52^d^	99.61±9.21^b^	65.39±2.62^c^	81.67±5.35^b^	124.96±16.23^d^	90.52±19.80^b^	156.33±13.04^e^
Triacylglycerols (mg/dl)	38.16±1.76^a^	31.28±2.23^b^	21.92±2.69^c^	38.17±3.08^a^	39.37±1.76^a^	29.09±2.39^b^	46.05±3.81^d^	43.51±2.72^e^
Phospholipid (mg/dl)	156.49±4.71^a^	180.63±22.36^b^	139.00±6.45^a^	166.21±11.64^a^	113.72±8.77^c^	173.99±4.61^b^	179.82±5.45^b^	133.16±25.93^a^
Free Fatty acid (mg/dl)	26.56±2.31^a^	35.72±0.78^b^	29.80±0.39^c^	29.06±1.25^c^	24.88±5.48^a^	30.30±1.14^c^	23.28±5.01^a^	31.48±7.36^c^
**Erythrocyte ghost**								
Cholesterol (mg/g)	7.03±0.27^a^	10.61±0.90^b^	6.93±0.24^a^	6.60±0.41^a^	6.45±0.33^a^	6.06±1.65^a^	6.18±0.34^a^	7.79±1.26^a^
Triacylglycerols (mg/g)	3.94±0.27^a^	6.84±0.68^c^	4.35±0.14^a^	2.81±0.29^a^	4.29±0.30^a^	4.21±1.20^a^	3.87±0.21^a^	5.15±0.82^b^
Phospholipid (mg/g)	7.91±0.26^a^	20.10±2.68^c^	10.12±0.22^b^	9.11±1.05^b^	13.43±0.69^b^	12.63±4.30^b^	12.08±0.68^b^	15.86±2.73^d^

Each value represents the mean±S.E.M of 5 rats. Values within the same row with different superscripts are significantly different at p<0.05.

The infection of the rats by *S. typhimurium* was associated with a significant (p<0.05) increase in erythrocyte ghost cholesterol, triacylglycerols and phospholipid concentrations. The increase in cholesterol amounted to 51% whereas increase in triacylglycerol and phospholipids amounted to 74% and 154% respectively. For cholesterol, the increase was reversed by both drugs on day 5 of treatment to a level not significantly (p>0.05) different from the normal control and the reversal was maintained through day 15. The response of triacyglycerol was also similar except for amoxillin on day 15 where the value obtained was higher than normal control but lower than the infection control. Although, there was a significant reversal of the elevated level of phospholipid by the chemotherapy, the levels produced by the treatments were still significantly (p<0.05) higher than the normal control.

Table 4 shows the effects of pefloxacin and amoxillin on cholesterol:phospholipid ratios in the plasma, HDL, LDL-VLDL, erythrocyte and erythrocyte ghost of rats infected with *S. typhimurium.* The infection of rats with *S. typhimurium* was associted with a significant (p<0.05) increase in the cholesterol:phospholipid ratios in the plasma, HDL, LDL-VLDL and erythrocyte ghost. The elevation of this ratio in the plasma was reversed in all the groups except infected+amoxicillin day 15. Similar reversals were observed in HDL and LDL-VLDL when compared with the infection control. Except for pefloxacin on day 10, amoxillin on days 10 and 15, the elevation of this ratio in the erythrocyte was not significantly (p>0.05) altered by any of the treatments.

**Table 4:** Cholesterol: Phospholipid ratios in the plasma, HDL, LDL-VLDL, erythrocyte and erythrocyte ghost of the animals

	**Normal control**	**Infection control**	**Infected+ pefloxacin day 5**	**Infected+ pefloxacin day 10**	**Infected + pefloxacin day 15**	**Infected+ amoxillin day 5**	**Infected+ amoxillin day 10**	**Infected + amoxillin day 15**
Plasma	0.87±0.16^a^	1.02±0.34^b^	0.69±0.10^a^	0.87±0.15^a^	0.72±0.04^a^	0.56±0.11^a^	0.70±0.10^a^	1.62±0.60^c^
HDL	0.57±0.04^a^	0.79±0.03^d^	0.38±0.09^b^	0.52±0.12^c^	0.43±0.06^b^	0.25±0.07^b^	0.66±0.14^c^	0.50±0.07^c^
LDL-VLDL	0.30±0.03^a^	0.51±0.06^c^	0.31±0.09^a^	0.42±0.07^b^	0.36±0.05^b^	0.41±0.06^b^	0.38±0.040^b^	0.41±0.03^b^
Erythrocyte	0.78±0.10^a^	0.71±0.12^a^	0.77±0.08^a^	0.39±0.02^b^	0.74±0.07^a^	0.75±0.15^a^	0.49±0.08^b^	1.53±0.50^c^
Erythrocyte ghost	0.89±0.04^a^	0.54±0.07^b^	0.68±0.02^b^	0.52±0.02^b^	0.48±0.01^b^	0.53±0.04^b^	0.51±0.02^b^	0.49±0.01^b^

Each value represents the mean ± S.E.M of 5 rats. Values within the same row with different superscripts are significantly different at p<0.05.

**Figure 1:**
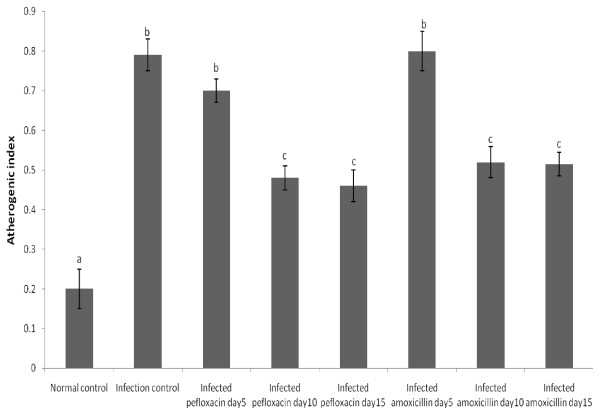
**Atherogenic indices of the animals.** Each bar represents the mean ± S.E.M of 5 rats. Bars with different alphabets are significantly different at p<0.05.

The effects of pefloxacin and amoxillin on atherogenic index of rats infected with *S. typhimurium* is depicted in Figure 1. Salmonellosis caused a 3 fold increase in this index. This was reduced by both pefloxacin and amoxillin after 10 days of treatment and this reversal was maintained by both drugs through day 15.

The summary of the effects of pefloxacin and amoxicillin on the lipid profiles of organs of rats infected with *S. typhimurium* is shown in Table 5. Salmonellosis caused a significant (p<0.05) increase in the levels of cholesterol and triacylglycerols in all the organs. However, phospholipid concentration was significantly (p<0.05) increased in the liver, kidney and brain alone with spleen and heart having decreased levels. In the liver, treatment with pefloxacin and amoxillin for 5 and 10 days caused a further significant (p<0.05) increase in cholesterol level. However, this increase was reversed in pefloxacin treated group on day 15. While the increase in hepatic triacylglycerols by both antibiotics was reversed on day 15, that of hepatic phosphoslipid was not significantly altered.

**Table 5:** Lipid profiles of liver, kidney, brain, spleen and heart of the animals

**Liver**	**Normal control**	**Infection control**	**Infected+ pefloxacin day 5**	**Infected+ pefloxacin day 10**	**Infected + pefloxacin day 15**	**Infected+ amoxillin day 5**	**Infected+ amoxillin day 10**	**Infected + amoxillin day 15**
Cholesterol (mg/g)	2.34±0.07^a^	2.57±0.03^b^	2.88±0.12^c^	2.62±0.08^d^	2.33±0.06^a^	2.71±0.12^d^	2.64±0.10^d^	2.72±0.08^d^
Triacylglycerols (mg/g)	1.56±0.19^b^	1.97±0.13^d^	2.39±0.08^e^	1.85±0.14^c^	1.22±0.07^a^	2.32±0.08^e^	2.22±0.07^e^	1.66±0.08^c^
Phospholipid (mg/g)	14.29±0.95^a^	27.30±0.92^c^	24.79±1.93^b^	26.15±3.82^b^	21.00±0.93^b^	25.17±1.69^b^	21.48±1.48^b^	23.72±1.31^b^
**Kidney**								
Cholesterol (mg/g)	2.58±0.35^a^	3.05±0.25^b^	2.48±0.38^a^	2.60±0.30^a^	2.44±0.32^a^	3.25±0.14^b^	4.33±0.62^c^	3.09±0.75^b^
Triacylglycerols (mg/g)	4.25±0.29^a^	9.35±0.31^c^	9.92±0.50^c^	12.24±0.57^d^	6.99±0.44^b^	6.23±1.03^b^	12.24±0.76^d^	6.71±0.67^b^
Phospholipid (mg/g)	10.01±0.79^a^	18.79±0.57^d^	11.57±0.85^a^	15.94±1.21^cd^	9.14±0.32^a^	12.25±1.08^a^	13.02±1.71^b^	9.72±1.12^a^
**Brain**								
Cholesterol (mg/g)	12.24±0.59^a^	19.72±1.23^c^	10.70±0.68^a^	16.05±0.86^b^	19.90±1.01^c^	16.29±1.28^b^	17.12±0.95^e^	16.72±1.43^e^
Triacylglycerols (mg/g)	0.60±0.06^a^	1.96±0.03^c^	1.97±0.03^c^	2.10±0.12^e^	2.08±0.05^e^	2.22±0.05^d^	1.91±0.04^f^	1.88±0.07^b^
Phospholipid (mg/g)	39.46±2.00^a^	49.17±2.28^b^	47.43±1.97^b^	42.38±2.03^b^	54.04±2.86^c^	52.59±3.80^c^	39.95±2.46^a^	38.01±4.63^a^
**Spleen**								
Cholesterol (mg/g)	1.09±0.11^a^	1.63±0.23^b^	1.47±0.23^c^	2.21±0.26^e^	2.12±0.06^e^	2.02±0.13^d^	1.43±0.33^c^	2.19±0.34^e^
Triacylglycerols (mg/g)	0.56±0.16^a^	1.50±0.07^b^	1.00±0.20^b^	1.04±0.18^b^	1.26±0.24^b^	1.57±0.32^b^	1.58±0.60^b^	1.96±0.50^c^
Phospholipid (mg/g)	12.49±1.35^a^	8.55±0.53^a^	9.28±0.98^d^	10.69±1.11^b^	8.41±0.37^c^	11.37±0.48^e^	9.23±0.47^d^	11.86±0.48^a^
**Heart**								
Cholesterol (mg/g)	0.58±0.05^a^	0.82±0.04^c^	0.82±0.04^c^	0.53±0.02^a^	0.41±0.01^b^	0.72±0.06^c^	0.41±0.02^b^	0.46±0.05^b^
Triacylglycerols (mg/g)	2.81±0.48^a^	4.63±0.34^a^	4.02±0.43^a^	4.04±0.75^a^	3.86±0.87^a^	4.71±1.17^a^	4.22±0.78^a^	3.51±0.69^a^
Phospholipid (mg/g)	10.35±0.46^a^	7.45±1.00^b^	8.12±1.10^c^	8.94±0.80^c^	7.82±0.52^c^	7.82±0.65^c^	7.53±0.43^b^	8.99±0.84^b^

Each value represents the mean±S.E.M of 5 rats. Values within the same row with different superscripts are significantly different at p<0.05.

In the kidney, treatment with pefloxacin resulted in a significant (p<0.05) decrease in cholesterol concentration from day 5 while amoxillin caused a further significant (p<0.05) increase. Although the increase in triacyglycerol was decreased on day 15 in infected rats treated with both antibiotics, their levels were still significantly (p<0.05) higher than that of the normal control. The phospholipid levels in infected rats treated with pefloxacin and amoxillin was still significantly (p<0.05) high on day 10; however, there was a significant (p<0.05) and complete reversal on day 15.

The decrease in the level of brain cholesterol after 5 days of pefloxacin and amoxillin administration was reversed on days 10 and 15. Both antibiotics caused further increase in the brain triacylglycerols after 5 days of administration with amoxillin treatment giving a reversal on days 10 and 15. Pefloxacin administration caused a further significant (p<0.05) increase in brain phospholipid level on day 15 alone while amoxillin therapy caused a significant (p<0.05) increase on day 5. However, this was reversed on day 10.

In the spleen, treatment of salmonellosis with pefloxacin caused a significant (p<0.05) decrease in cholesterol and triacyglycerol levels. As treatment continued however, there was a rebound to higher values in both cholesterol and triacyglycerol. The salmonellosis-induced decrease in the spleen phospholipid was reversed by both drugs on day 5 and by day 15 amoxillin-treated rats had phospholipid concentration not significantly (p>0.05) different from that of the normal control.

Salmonellosis-induced cardiac hypercholesterolemia was reversed after 10 days of treatment with both antibiotics. Both antibiotics caused a further significant (p<0.05) increase in heart phospholipid level from day 5 through day 15 except amoxillin day 10 which was not significantly (p>0.05) different from the normal control.

Table 6 shows the summary of the effects of pefloxacin and amoxillin on the cholesterol:phospholipid ratios in the organs of rats infected with *S. typhimurium.* Salmonellosis caused a significant (p<0.05) decrease in this ratio in liver and kidney with a significant (p<0.05) increase in brain, spleen and heart. The decrease in the liver and kidney was reversed after treatment with both antibiotics. Both drugs only caused a significant (p<0.05) decrease of the elevated cholesterol:phospholipid ratio in brain on day 5 of the treatment. In the spleen, the values of this ratio in all the treatments were not significantly (p>0.05) different from the infection control except infected+amoxicillin day 10 which was significantly (p<0.05) lower than the infection control. On day 10 of treatment with both antibiotics, the elevation observed in the heart was reversed to a level not significantly (p>0.05) different from the normal control.

**Table 6:** Cholesterol: Phospholipid ratios in the organs of the animals

	**Normal control**	**Infection control**	**Infected+ pefloxacin day 5**	**Infected+ pefloxacin day 10**	**Infected + pefloxacin day 15**	**Infected+ amoxillin day 5**	**Infected+ amoxillin day 10**	**Infected + amoxillin day 15**
Liver	0.17±0.01^a^	0.09±0.01^c^	0.12±0.01^b^	0.11±0.02^b^	0.11±0.01^b^	0.110±0.01^b^	0.13±0.01^b^	0.12±0.01^b^
Kidney	0.28±0.06^a^	0.16±0.02^b^	0.21±0.04^a^	0.19±0.04^a^	0.27±0.04^c^	0.259±0.02^a^	0.34±0.04^c^	0.31±0.06^c^
Brain	0.31±0.02^a^	0.40±0.02^b^	0.26±0.04^c^	0.38±0.01^b^	0.37±0.02^a^	0.313±0.01^c^	0.43±0.02^d^	0.46±0.07^d^
Spleen	0.09±0.02^a^	0.18±0.03^b^	0.18±0.03^b^	0.21±0.02^b^	0.25±0.01^b^	0.175±0.01^b^	0.16±0.04^c^	0.19±0.03^b^
Heart	0.06±0.01^a^	0.12±0.02^b^	0.12±0.02^b^	0.06±0.01^a^	0.05±0.01^c^	0.095±0.01^b^	0.06±0.01^a^	0.05±0.01^a^

Each value represents the mean±S.E.M of 5 rats. Values within the same row with different superscripts are significantly different at p<0.05.

**Figure 2:**
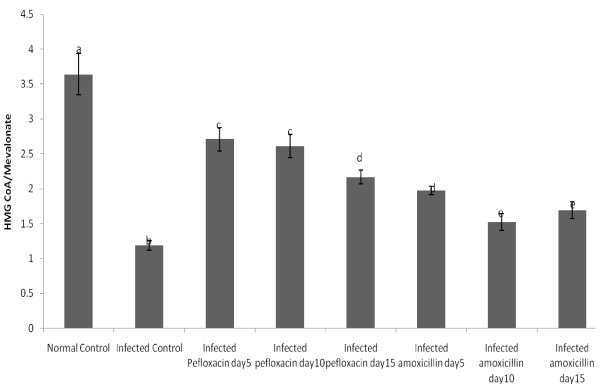
**Hepatic HMG CoA/Mevanolate ratios of the animals.** NB: This ratio is used as an index of HMG CoA reductase activity. Each bar represents the mean±S.E.M of 5rats. Bars with different alphabets are significantly different at p<0.05.

Figure 2 depicts the effects of salmonellosis and its chemotherapy with pefloxacin and amoxillin on hepatic HMG-CoA/mevalonate ratios as an index of the activity of HMG-CoA reductase. Salmonellosis caused a 3.5 fold decrease in this ratio. Both antibiotics reversed this trend. The reversal by pefloxacin was however more pronounced than that of amoxillin. Five days after the withdrawal of both antibiotics, activity of HMG-CoA reductase had not returned to control values.

Intensity of associations between fecal bacteria load and lipid profiles and hepatic HMG-CoA/Mevalonate ratio in rats infected with *S. typhimurium* is shown in Table 7. The fecal bacteria load was positively correlated with plasma cholesterol (r=0.456, p<0.01), plasma triacylglycerols (r=0.485, p<0.01), plasma phospholipid (r=0.414, p<0.01), plasma free fatty acid (r=0.485, p<0.01), LDL-VLDL triacylglycerols (r=0.313, p<0.05), liver cholesterol (r= 0.464, p<0.01), liver triacylglycerols (r= 0.546, p<0.01), liver phospholipid (r= 0.680, p<0.01), kidney triacylglycerols (r=0.445, p<0.01), kidney phospholipid (r= 0.459, p<0.01), brain triacylglycerols (r=0.785, p<0.01), brain phospholipid (r= 0.343, p<0.05), heart cholesterol (r= 0.488, p<0.01) and erythrocyte ghost phospholipid (r=0.359, p<0.05). A negative association was however observed with HDL cholesterol (r= −0.485, p<0.01), erythrocyte triacylglycerols (r= −0.318, p<0.05), spleen phospholipid (r= −0.419, p<0.01), heart phospholipid (r= −0.415, p<0.01) and hepatic HMG CoA/Mevalonate ratio (r= −0.501, p<0.01).

**Table 7:** Intensity of association between fecal bacteria load and lipid profiles and hepatic HMG CoA/Mevalonate ratio in the animals

**Parameters**	**Correlation coefficient**
Fecal Bacteria load vs. Weight change	−0.609^a^
Fecal Bacteria load vs. Plasma Cholesterol	0.456^a^
Fecal Bacteria load vs. Plasma Triacylglycerols	0.485^a^
Fecal Bacteria load vs. Plasma Phospholipid	0.414^a^
Fecal Bacteria load vs. Plasma free fatty acids	0.485^a^
Fecal Bacteria load vs. HDL Cholesterol	−0.485^a^
Fecal Bacteria load vs. LDL-VLDL Triacylglycerols	0.313^b^
Fecal Bacteria load vs. Erythrocyte Triacylglycerols	−0.318^b^
Fecal Bacteria load vs. Liver Cholesterol	0.464^a^
Fecal Bacteria load vs. Liver Triacylglycerols	0.546^a^
Fecal Bacteria load vs. Liver Phospholipid	0.680^a^
Fecal Bacteria load vs. Kidney Triacylglycerols	0.445^a^
Fecal Bacteria load vs. Kidney Phospholipid	0.459^a^
Fecal Bacteria load vs. Brain Triacylglycerols	0.785^a^
Fecal Bacteria load vs. Brain Phospholipid	0.343^b^
Fecal Bacteria load vs. Spleen Phospholipid	−0.419^a^
Fecal Bacteria load vs. Heart Cholesterol	0.488^a^
Fecal Bacteria load vs. Heart Phospholipid	−0.415^a^
Fecal Bacteria load vs. Erythrocyte ghost Phospholipid	0.359^b^
Fecal Bacteria load vs. HMG CoA/Mevalonate	−0.501^a^

a Correlation is significant at p< 0.01.

b Correlation is significant at p< 0.05.

## Discussion

The results of this study indicate that fecal excretion of salmonella and weight loss are associated with salmonella infection and that antibiotics may not completely eradicate salmonella from the colon. Our results are consistent with other studies that have reported colonisation of both the ileum and colon by the microbe and subsequent fecal excretion for several weeks [18]. Weight loss in salmonella infection, attributed to diarrhea, decrease in appetite and general inflammation of the gastrointestinal tract, have also been reported [18,19].

A major finding of this study was that Salmonellosis perturbs the metabolism of lipids in different compartments of the animals. These perturbations were reflected as up-/down-regulation of the concentrations of the major lipids (cholesterol, triacylglycerols, phospholipids and free fatty acids). The data also indicate that the activity of hepatic HMG-CoA reductase was up-regulated as a result of salmonellosis and its chemotherapy. During infection, a wide range of alterations in metabolism occur. These are part of the body’s reactions which help protect the host from further injury, and facilitates the repair process [20]. However, these metabolic changes, if present for prolonged periods, can lead to detrimental consequences on the host.

The increased triacylglycerol levels as well as increase in VLDL and reduced HDL cholesterol levels observed in this study are characteristic changes that have been reported to occur during infection [21,22]. The consequence of this type of change is promotion of atherogenesis as observed by increased atherogenic index of the animals. Elevation of plasma triacylglycerol level could be the result of either increased VLDL production or decreased VLDL clearance [23,24]. Experiments in rats and mice showed that even at low doses, bacteria LPS can rapidly stimulate VLDL production by increasing adipose tissue lipolysis, increasing hepatic *de novo* fatty acid synthesis, and decreasing hepatic fatty acid oxidation, all of which provide fatty acid substrate for esterification into triacylglycerols and assembly into VLDL particles in the liver [24-27]. Injection of bacteria or LPS into rats had been reported to significantly inhibit the clearance of LDL from the circulation [28] and markedly increase VLDL-triacylglycerol [29,30]. This mechanism may also have been responsible for the increased plasma and erythrocyte free fatty acids observed in the experimental rats infected with *Salmonella* as the increase in free fatty acids was associated with increased LDL-VLDL and plasma triacylglycerols.

The findings of this study showed a salmonellosis-induced hypercholesterolemia in the tissues of the experimental animals. This enhanced cholesterogenesis in the liver and brain of the animals may be attributed to salmonella-induced activation of HMG-CoA (the rate-limiting enzyme in cholesterol synthesis), while the accumulation of cholesterol by other extra-hepatic tissues may be mediated by an enhanced cholesterol efflux from the liver since most extra-hepatic tissues obtain their cholesterol from the liver [31]. This also suggests inhibition of the enzyme cholesterol-7α-hydroxylase, the rate-limiting enzyme in the conversion of cholesterol to bile acids (a well known mechanism for the excretion of cholesterol from the body) [32].

Another mechanism of cholesterol removal from the tissues (most especially extra-hepatic tissues), is the apolipoprotein-mediated cholesterol removal. An impairment of this process is usually indicated by decreased HDL cholesterol level [33] as observed in this study. HDL and apolipoprotein (apo) A-I have been reported to be receptors of cellular cholesterol [33]. In addition, phospholipid transfer protein mediates a transfer of phospholipids and cholesterol between triacylglycerols-rich lipoproteins and HDL, and a reduction in phospholipid transfer protein activity results in lower HDL levels [33]. Therefore, a reduction in phospholipid transfer protein activity could presumably decrease cellular cholesterol removal as well. Other factors that influence the level of tissue cholesterol are the activities of plasma lecithin:cholesterol acyltransferase (LCAT) and cholesterol ester transfer protein (CETP) [34]. While LCAT is required for the conversion of free cholesterol into cholesterol ester so that it can then move into the core of HDL, CETP mediates the exchange of cholesterol ester in HDL for triacylglycerols in triacylglycerol-rich lipoproteins, an important step in the delivery of cholesterol to the liver from extra-hepatic tissues [34]. Although the activities of the two enzymes were not determined in this study, reductions in their activities which have been reported during infection in other studies [35,36] could retard the reverse cholesterol pathway as observed in this study.

The findings of this research also demonstrated a salmonellosis-induced phospholipidosis in the experimental animals. The induction of phospholipidosis is often characterized by: (1) inhibition of lysosomal phospholipase activity—this is generally regarded as the primary mechanism of induction, (2) inhibition of lysosomal enzyme transport as a result of down-regulation of genes involved in lysosomal enzyme transport, (3) enhanced phospholipid biosynthesis due to enhanced free fatty acid availability and (4) enhanced cholesterogenesis [37]. The data from this study indicate that the latter two mechanisms might be involved in the induction of phospholipidosis. Previous studies have shown that an infusion of a phospholipid-rich lipid emulsion improved survival in a porcine model of septic peritonitis [29,30], hence plasma, erythrocyte and tissue phospholipidosis observed in this study might possibly be part of the host response to salmonellosis. The ability of oxidized phospholipids to modulate the LPS signaling is known to be beneficial to the host during infection. In fact, oxidized phospholipids have been shown to decrease inflammatory process in mice treated with LPS, protecting them from endotoxic shock [36]. This may explain why treatment with the antibiotics was unable to reverse phospholipidosis as these antibiotics are known to cause the release of LPS from the bacteria cells into circulation [36].

## Conclusion

Results of the present study provide evidence that dyslipidemia during and after administration of both amoxiliin and pefloxacin does occur in rat model of salmonellosis. If these metabolic changes are present for prolonged periods, they could be early signs in the pathophysiology of atherosclerosis.

## Methods

### Chemicals

Pefloxacin was a product of Lek Pharmaceutical and Chemical Company, Ljubljana, Slovenia, while amoxillin was obtained from Beecham Pharmaceuticals, Brentford, England. All other chemicals used in this study were of the purest grade available and were obtained from British Drug House (BDH) Chemicals Limited, Poole, England and Sigma-Aldrich, Missouri, U. S. A.

### Bacteria strain

*Salmonella enterica* serovar *Typhimurium strain* TA98 (obtained from the Nigerian Institute of Medical Research (NIMR), Yaba, Lagos, Nigeria) was grown for 48 h under static conditions in nutrient broth (CMI, Unipath, UK). The organism was maintained on nutrient agar slant at 4°C. Bacteria were harvested from the slant, suspended in 100ml nutrient broth and allowed to grow at 37°C for 12 h (late logarithmic growth phase). The cells were spun at 4,300 × g at 4°C for 10 min. Once the supernatant was discarded, the cells were resuspended in 20 ml of buffer and the centrifugation was repeated. The final washed pellet of bacteria cells was resuspended in 10 ml of phosphate-buffered saline (PBS) pH 7.4. The stock concentration of bacteria was determined from an optical density curve with a spectrophotometer.

### Animal handling and experimental protocol

Experimental protocols were conducted in accord with guidelines of the Institutional Animal Care and Use Committee and were approved by the Animal Ethical Committee of the Department of Biochemistry, University of Agriculture Abeokuta. Pathogen free male albino rats weighing 230-280 g obtained from the NIMR were used for the study. The rats were housed in metabolic cages at room temperature (22-24°C) and had free access to rat chow and autoclaved tap water throughout the period of the experiment. Animals were acclimatized to the housing and dietary conditions for 2 weeks after which they were fasted overnight and infected orally with 0.2 ml phosphate-buffered saline (PBS) pH 7.4 containing 1 × 10^10^ colony-forming unit (CFU) live culture of the bacteria as described Hung and Wang [38]. Twenty animals that were not infected and received 0.2 ml PBS orally served as the normal control.

In previous *Salmonella* infection studies in rats [18,21] it was established that monitoring functional infection outcomes like *Salmonella* colonisation, translocation and infection induced changes, follow-up of infected rats for at least 3 to 4 days is needed. Therefore the infected rats were left for four days after which fresh faecal samples were collected to quantify *Salmonella* colonisation daily, as described by van Ampting [39].

After salmonellosis was confirmed, infected animals were divided into 7 groups of 5 animals each. While 1 group served as infection control group and sacrificed immediately to obtain baseline values in salmonellosis (Infection Control), three groups were treated with amoxillin (7.14 mg/kg body weight, 8 hourly) and the remaining three groups with pefloxacin (5.71 mg/kg body weight, 12 hourly) for 5 and 10 days respectively. The antibiotics were constituted in 5% dextrose and were prepared fresh before each administration. They were administered in a total volume of 0.1ml. Control animals received equivalent volume of 5% dextrose. All drug administration was by the intraperitoneal route. Table 8 summarises the study protocol.

**Table 8:** Study protocol

**Treatment Group**	**Treatment**
Control Group 1	Control animals sacrificed when treatment of salmonella-infected animals with antibiotics commenced (Designated as **Normal Control** in Tables)
Control Group 2	Control animals for 5 days of antibiotic treatment
Control Group 3	Control animals for 10 days of antibiotic treatment
Control Group 4	Control animals for day 15 (5 days after discontinuation of antibiotic treatment)
Salmonella group 5	Infected animals sacrificed when treatment with antibiotics commenced (Designated in Tables as **Infection Control**)
Salmonella group 6	Infected animals treated with amoxillin for 5 days and sacrificed 24h after amoxillin treatment (Designated in Tables as**Infected+Amoxillin day 5**)
Salmonella group 7	Infected animals treated with amoxillin for 10 days and sacrificed 24h after amoxillin treatment (Designated in Tables as **Infected+Amoxillin day 10**)
Salmonella group 8	Infected animals treated with amoxillin for 10 days and left for another 5 days to recover from antibiotic treatment before being sacrificed (Designated in Tables as **Infected+Amoxillin day 15**)
Salmonella group 9	Infected animals treated with pefloxacin for 5 days and sacrificed 24h after pefloxacin treatment (Designated in Tables as **Infected+Pefloxacin day 5**)
Salmonella group 10	Infected animals treated with pefloxacin for 10 days and sacrificed 24h after pefloxacin treatment (Designated in Tables as **Infected+Pefloxacin day 10**)
Salmonella group 11	Infected animals treated with pefloxacin for 10 days and left for another 5 days to recover from antibiotic treatment before being sacrificed (Designated in Tables as **Infected+Pefloxacin day 15**)

Twenty four hours after 5 and 10 days antibiotic treatment and 5 days after the discontinuation of the antibiotics, blood was collected from the animals into heparinised tubes by cardiac puncture under light ether anaesthesia after an overnight fast. Their corresponding controls were also sacrificed at the same time point. Liver, kidney, brain, heart and spleen were removed from the animals for biochemical analyses. Blood samples were centrifuged to separate plasma and red blood cells. All samples were stored at −20°C until analysed. Since the values for many of these parameters in control animals did not change appreciably during the course of the study, only values for one group of ‘Normal Control’ are presented in the tables.

### Biochemical analyses

#### Plasma lipid profiles

Plasma concentrations of total cholesterol and triglycerides were determined with commercial kits (Spin React S.A., Santa Colona, Sant Esteve De Bas, Spain). HDL cholesterol and triglycerides were determined in plasma with same commercial kits for total cholesterol and triglycerides after very low density lipoproteins (VLDL) and LDL were precipitated with heparin-MnCl_2_ solution [40]. Total phospholipids in plasma were extracted with chloroform-methanol mixture (2:1, v/v) as described by Folch et al. [41]. Phospholipid content was then determined as described by Stewart [42]. Briefly, an aliquot of the phospholipid extract was evaporated to dryness at 60°C. After cooling, 2 ml of chloroform was added to the dried lipid extract and vortexed. Ammonium ferrothiocyanate (2 ml) was then added and the mixture vortexed for 1 min. They were left for 10 min for the phases to separate. The chloroform layer was taken and absorbance read at 488 nm. Phospholipid concentrations were then determined using a phospholipid standard as reference.

Atherogenic Index (AI) was calculated as the base 10 logarithm of the ratio of the concentration of triacyglycerol to high density lipoprotein cholesterol, where each concentration is expressed in mmol/L [43].

Free fatty acids (FFA) in plasma were determined according to the method of Soloni and Sardina [44] as modified by Brunk and Swanson [45]. Briefly, to 100 μl of plasma was added 300 μl of copper reagent and 2 ml of chloroform. This was shaken with a vertical shaker for 10 min and centrifuged. After centrifugation, the chloroform layer was removed and to this was added 1 ml of cuprizone and 100 μl of ammonia reagent. The contents were shaken briefly by hand and absorbance read at 620 nm 10 min after adding ammonia reagent. A standard curve of palmitic acid taken through the same procedure was used to calculate the concentrations of FFA in the plasma samples.

#### Erythrocyte lipid profile

Because the Folch extraction [41] produced lipid extracts which were highly pigmented, an improved procedure for the extraction of lipids from erythrocytes using chloroform-isopropanol (7:11, v/v) described by Rose and Oklander [46] was employed. For the determination of cholesterol, an aliquot of the chloroform-isopropanol extract was evaporated to dryness at 60°C. Triton X-100/chloroform mixture (1:1, v/v, 20 μl) was added to resolve the lipids and again the solvent was evaporated. Then 1 ml of commercially available cholesterol kit reagent (Spin React S.A., Santa Colona, Sant Esteve De Bas, Spain) was added and vortexed. After incubation in the dark at room temperature for 30 min, cholesterol content was determined by colorimetry [47]. Determination of total phospholipids and free fatty acids in the chloroform-isopropanol extract of the erythrocyte followed the same procedure as described for plasma [42].

#### Organ lipid profiles

Lipids were extracted from the organs (liver, kidney, brain, heart and spleen) as described by Folch et al. (1957). After washing with 0.05M KCl solution, aliquots of the chloroform-methanol extract were then used for the determination of cholesterol, triglycerides and phospholipids concentrations. Cholesterol was determined in an aliquot of the chloroform-methanol extract of each organ as described for erythrocytes while determination of phospholipids followed the same procedure as described for plasma. Triglyceride concentrations in aliquots of the chloroform-methanol extracts of each organ were determined following the procedure described by Kriketos et al. [48]. Briefly, an aliquot of the chloroform-methanol extract in Eppendorf tubes was evaporated to dryness at 60°C. After cooling, 200 μl of ethanol (97%) was added to the tube to resuspend the triglyceride. Then 1 ml of commercially available triglyceride kit (Spin React S. A., Santa Colona, Sant Esteve De Bas, Spain) was added and vortexed. After incubating in the dark at room temperature for 20 min, triglyceride content was determined spectrophotometrically.

#### Isolation of erythrocyte ghost and determination of its lipid profile

Erythrocyte membranes were prepared according to the method described by Hanahan and Ekholm [49]. Briefly, blood samples were centrifuged at 5000 rpm for 15 min at 4°C. Plasma and buffy coat were removed by careful suction and the cells were resuspended in isotonic Tris–HCl buffer. After mixing by inversion, the samples were recentrifuged at 5000 rpm for 15 min at 4°C. The supernatant was again removed by careful suction and a few red cells were sacrificed to remove any remaining buffy layer. This washing procedure was repeated twice. The washed cells were then suspended in isotonic Tris–HCl buffer pH 7.6 to an approximate hematocrit of 50% and were kept on ice. The samples were mixed gently by inversion for about 1 min before membrane preparation. 5ml aliquots of the 50% cell suspensions were transferred to 50 ml polyethylene tubes. Thirty ml of hypotonic Tris–HCl buffer pH 7.6 were added to the cell suspension for osmotic lysis. After allowing the tubes to stand for about 10 min, they were centrifuged at 20,000 rpm for 20 min at 4°C. The supernatants were discarded and the pellets resuspended in 10 ml Tris–HCl and centrifuged for 20 min at 20,000 rpm at 4°C. The pellets were washed four times until the membranes were colourless. Finally, the resultant pellets were rinsed twice with 100 μl cold Tris–HCl buffer and poured into Eppendorf tubes. The membrane suspensions were kept frozen in this latter buffer at −20°C. Lipids were extracted from the membrane suspensions and determined as described for erythrocytes.

### Determination of hepatic HMG-CoA reductase activity

This was determined according to the method of Rao and Ramakrishnan [50]. Briefly, fresh liver homogenates were mixed with equal volumes of perchloric acid. The mixture was subsequently centrifuged (2,000 rpm for 10 min) and the supernatant was treated with freshly prepared 1M hydroxylamine in 2.25M NaOH. HMG-Co A was determined by subsequent colorimetric measurement of the resulting hydroxamic acid by formation of complexes with ferric salts at 540 nm. Mevalonate was estimated by reaction with 1M hydroxylamine in water to form the hydroxamate. The ratio of HMG-CoA to mevalonate is taken as an index of the activity of HMG-CoA reductase. An increase in this ratio indicates inhibition of cholesterogenesis while a decrease indicates enhanced cholesterogenesis.

### Statistical analysis

Data are expressed as mean ± S.E.M. One way analysis of variance (ANOVA) followed by Duncan Multiple Range Test was used to analyse the results with p < 0.05 considered significant.

## Competing interests

The authors declare that they have no competing interests.

## Authors’ contributions

RSO carried out literature search and all experimental work, performed statistical analysis and data interpretation and wrote the draft of the manuscript. ODA contributed to conception and design, analysis and interpretation of data and critical review of the manuscript. TOA contributed to conception and design, analysis and interpretation of data and critical review of the manuscript. BEA contributed to conception and design, analysis and interpretation of data and critical review of the manuscript. AO supervised the work and contributed intellectual input in the discussion and overall presentation of the manuscript. All authors read and approved the final manuscript.
